# Photochemical Treatment of *Drosophila* APCs Can Eliminate Associated Viruses and Maintain the APC Function for Generating Antigen-Specific CTLs Ex Vivo

**DOI:** 10.1155/2018/4167652

**Published:** 2018-09-20

**Authors:** Jun Ye, Chunxia Yang, Zeling Cai, Weixing Shi, Hong Yu

**Affiliations:** ^1^Department of Laboratory Medicine, Taizhou People's Hospital, Taizhou, Jiangsu Province, China; ^2^Shanghai Yuyan Cell Research Company, 3F Building 4 No. 879 Zhongjiang Road, Shanghai, China; ^3^Jiangsu CTL Biological Technology Co. Ltd., 9F, G25, Koutai East and Xinyang North Crossroad, CMC, Taizhou, Jiangsu, China; ^4^Johnson & Johnson Pharmaceutical Research and Development, LLC, 3210 Merryfield Row, San Diego, CA 92121, USA

## Abstract

*Drosophila* cells transfected with MHC class I and a number of costimulation molecules including B7.1, ICAM, LFA-3, and CD70 are potent antigen-presenting cells (APCs) for the generation of antigen-specific cytotoxic T cells (CTLs) *in vitro*. Using *Drosophila* APCs, CTLs specific for melanoma antigens have been generated *in vitro* and adoptively transferred to melanoma patients. However, the recent discovery that *Drosophila* cells can carry insect viruses raises the potential risk of *Drosophila* APCs transmitting xenogenic viruses to patient CTLs. In this study, we have investigated photoreactive methods to inactivate insect viruses in APC. A clinical grade psoralen compound, 8-MOP (UVADEX) in combination with UVA treatment (5 joules/cm^2^) can be used to inactivate *Drosophila* cell viruses. UVADEX treatment is sufficient to inactivate insect viruses but does not affect the expression of MHC class I molecules and costimulation molecules on *Drosophila* APCs. In fact, UVADEX treatment prevents *Drosophila* APC growth while maintaining APC function. Furthermore, UVADEX-treated *Drosophila* APCs maintain or have enhanced APC function as determined by enhanced T cell activation, proliferation, and CTL generation. Thus, the use of UVADEX-treated *Drosophila* APCs may provide a valuable tool for immunotherapy to generate tumor antigen-specific CTLs.

## 1. Introduction

Cancer immunotherapy, a type of treatment that pushes the immune system to attack tumors, has been ranked at the top of the list of scientific achievements in 2013 [1]. An adoptive cell immunotherapy, otherwise known as activated T cell therapy, has been developed to treat cancer [2, 3]. Adoptive cell immunotherapy involves activation of the patient's own T cells to generate cytotoxic T lymphocytes (CTLs) which can kill tumor cells specifically. CTLs are activated ex vivo by exposing naïve CD8^+^ T cells to antigenic peptide/MHC complexes presented by antigen-presenting cells (APCs) [4]. The binding between TCR on CD8 T cells and peptide/MHC complexes on APC leads to T cell proliferation and differentiation. Dendritic cells, macrophages, and B cells can all function as APCs. In addition to MHC, the expression of several costimulatory molecules on APC is also crucial for T cell activation. Once CD8^+^ T cells are activated, they are differentiated into armed CTLs. The armed CTLs are then able to recognize and kill antigen-expressing target cells, such as virus-infected or cancer cells. Traditional antigen-presenting cells can be replaced by artificial antigen-presenting cells for the purpose of activating resting CD8^+^ T cells into CTLs [5, 6]. Insect cells, for example, *Drosophila* cells that are transfected with MHC class I and costimulatory and adhesion molecules, present a high density of peptides/MHC complexes and have been demonstrated as an effective APC system to stimulate naïve CD8 T cells and drive them to develop into effector cells with cytotoxic activity against target cells [7]. Cytotoxicity is specific to the antigen(s) to which the CTLs were immunized against *in vitro*.

As more treatments using interspecies biological materials are used, the safety of the patient is of primary concern. The recent discovery that *Drosophila* cells can carry insect viruses raises the potential risk of *Drosophila* APCs transmitting viruses to patient CTLs [8]. Current approaches including germicidal ultraviolet radiation, gamma irradiation, beta-propiolactone, alcohol, detergents, aldehydes, alkylating agents, heat, and other treatments to inactivate viruses may potentially alter APC function and CTL generation [9]. These current approaches do not, for example, preserve the native antigenicity, immunogenicity, and cell membrane integrity that is required for antigen-presenting cell function. As an alternative to these approaches, psoralen derivatives and long-wave ultraviolet light treatment can photo-react and irreversibly cross-link viral nucleic acids inside antigen-presenting cells, eliminating viral infectivity [10] while leaving surface molecules relatively unmodified. Psoralens are planar tricyclic compounds consisting of a furan ring fused to a coumarin moiety, furocoumarin [11]. Psoralen is a photochemical drug, which intercalates between the bases of double-stranded regions of DNA and RNA. When ultraviolet A light is absorbed, psoralen makes mono- and diadducts with pyrimidine bases in nucleic acid. Monoadducts and diadducts prevent subsequent nucleic acid replication of both host and pathogen nucleic acids. This thereby eliminates the infectivity of the viruses present in APCs. It has completed phase III clinical studies in the US and Europe for the safety of apheresis platelets with a photochemical process for pathogen inactivation [12].

Here, we demonstrated that UVADEX (8-MOP) treatment, combined with UV-irradiation, is able to inactivate known insect viruses and that UVADEX treatment does not decrease APC function. We generated antigen-specific CTLs by using *Drosophila* APCs treated with 8-MOP psoralen (UVADEX) which is a member of the psoralen family and long wavelength UVA light ex vivo. Our data show that UVADEX treatment prevents *Drosophila* cell growth, while cell surface molecule expression is unchanged. The ability of *Drosophila* cells to activate T cell, induce T cell proliferation, and generate cytotoxic function remains intact following UVADEX treatment. In addition, UVADEX plus UVA treatment inactivated insect viruses by preventing their replication. In this study, we demonstrated that photochemical treatment of *Drosophila* antigen-presenting cells with UVADEX and UVA ex vivo can eliminate the infectivity of infectious agents and maintain or increase APC function. Thus, UVADEX-treated *Drosophila* cells may be a useful reagent in developing cancer immunotherapy.

## 2. Results

### 2.1. Effect of Glutaraldehyde Fixation of *Drosophila* Cells on APC Function

**Figure 1:**
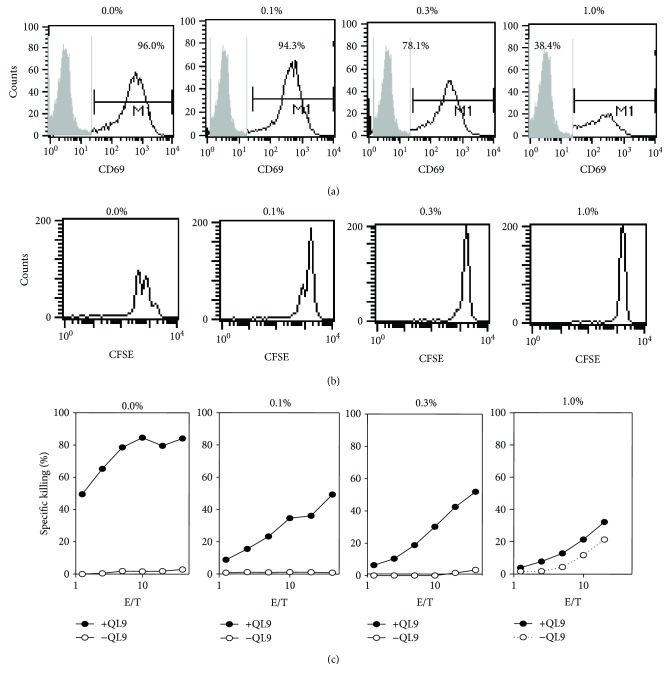
The effect of glutaraldehyde fixation of Fly/L^d^/B7.1/ICAM cells on CD8 T cell activation, proliferation, and CTL function. 1 mM CuSO_4_-induced Fly/L^d^/B7.1/ICAM cells were fixed in 2.5 ml of PBS containing 1%, 0.3%, 0.1%, and 0% glutaraldehyde at 10 × 10^6^/ml at RT for 30 mins, respectively. After completely washing with *Drosophila* media, the fixed cells were resuspended in 10 ml of MLR (mixed lymphocyte reaction) culture media, one part of cells was loaded with 10 *μ*M QL9 peptide at RT for 60 mins. (a) For cell activation, 1 × 10^6^ of purified mouse CD8 T cells were incubated with 1 × 10^6^ of QL9 loaded-fixed Fly cells at 37°C, 5% CO_2_ for 16 h. Cells were collected and stained with FITC-conjugated anti-mouse CD69 mAb and antibody isotype control at 4°C for 30 minutes. CD69 expression on CD8 cells was analyzed by FACS. (b) For cell proliferation, CD8 cells were labeled with 10 *μ*M CFSE at 37°C for 15 min. After complete washing with MLR media, 1 × 10^6^ of labeled cells were incubated with 1 × 10^6^ of QL9 loaded-fixed Fly cells at 37°C, 5% CO_2_ for 48 h. Cells were collected and stained with PE-conjugated anti-mouse CD8 mAb at 4°C for 30 min. CD8-positive cells were gated from FACS and further analyzed for their green florescence intensity. (c) For the measurement of CTL activity, CD8 T cells purified from three mixtures of spleen cells of 2c transgenic mice were stimulated with QL9 loaded-fixed Fly cells at 37°C, 5% CO_2_ at day 1, and then propagated in IL-2-supplemented media for 7 days. CTL activity was measured at day 7 by using ^51^Cr release assay with peptide-pulsed RmAsL^d^ target cells (closed dot lines). RmAsL^d^ cells without QL9 loading in the assay were a negative control (open dot lines).

Previous studies have used *Drosophila* cells transfected with MHC class I molecule and costimulatory molecules as artificial APCs to generate antigen-specific CTLs for immunotherapy [7]. However, the discovery that *Drosophila* cells can carry insect viruses raises the potential risk of *Drosophila* APCs transmitting viruses to patient CTLs. The viral-like particles were identified as *Drosophila* X virus (DXV) which is a lytic virus, *Drosophila* nodavirus (DrNV) and hantavirus pulmonary syndrome-1- (HPS-1-) like virus [8]. Therefore, inactivation of *Drosophila* viruses is a very important safety concern for CTL immunotherapy using *Drosophila* APCs. The inactivation of viruses using 0.1% of glutaraldehyde fixation has been reported before [13, 14]. The method of glutaraldehyde crosslinking [15] was also initially used to inactivate *Drosophila* viruses in *Drosophila* cells. To determine if glutaraldehyde has an effect on *Drosophila* APC function, CD69 expression, proliferation, and cytotoxicity of CD8 T cells stimulated by glutaraldehyde-fixed *Drosophila* APC were assayed. As shown in Figure 1(a), CD69 expression of CD8 T cells stimulated with QL9 loaded-fixed Fly cells was decreased with the increased amounts of glutaraldehyde comparing to the antibody isotype control staining (gray area on the left). In addition, increased glutaraldehyde concentration of caused decreased CTL proliferation and cytotoxicity (Figures 1(b) and 1(c)). All results were repeated in another respective experiment (data not shown) suggesting that although glutaraldehyde would presumably prevent *Drosophila* viral transmission, it is not an effective treatment because it diminishes *Drosophila* APC function.

### 2.2. UVADEX Treatment Inactivates *Drosophila* Viruses

**Figure 2:**
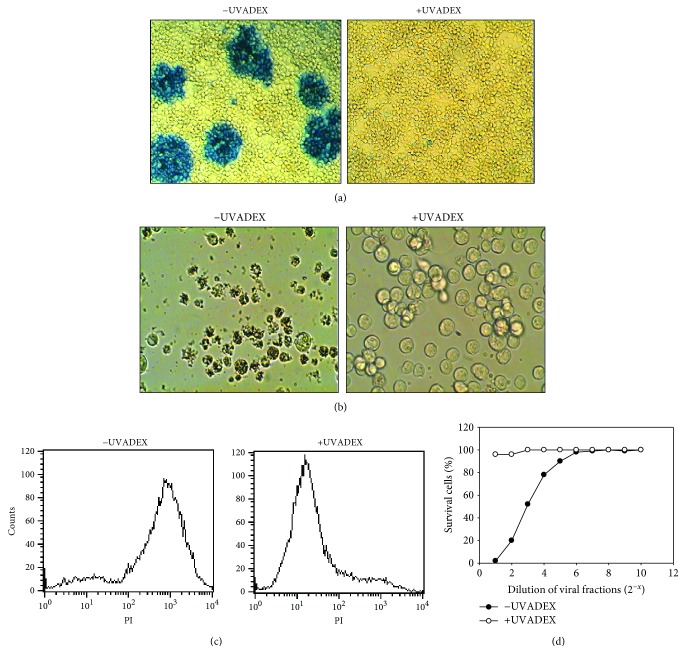
UVADEX treatment inactivates insect and *Drosophila* viruses. (a) Baculovirus-infected Sf9 cells were treated with UVADEX for 5 min or left untreated and grown for 4 days. Supernatant from infected cells was collected and used to reinfect Sf9 cells in a 96-well plate. Virus was detected by gp64 antibody staining using a rapid microtiter assay kit (BD BacPAKTM Baculovirus Rapid Titer Kit). Infected cells were pictured under a microscope. Left panel: viral infected cells. Right panel: UVADEX pretreated viral infected cells. (b) Inactivation of virus in *Drosophila* virus X-infected clone 5-5 cells by UVADEX. *Drosophila* viral-free cell line clone 5-5 was infected with UVADEX-pretreated or non-treated *Drosophila* viral fraction at RT for 1 h. The infected cells were washed with PBS completely to remove any residual viral particles and then cultured in media for 3 days. Infected cells were pictured under a microscope. Left panel: viral infected non-UVADEX-treated cells. Right panel: UVADEX-pretreated viral infected cells. (c) PI staining of UVADEX-pretreated or UVADEX-nontreated *Drosophila* viral fraction-infected clone 5-5 cells at day 3. (d) *Drosophila* lytic viral-free cell line clone 5-5 was infected with different dilutions of *Drosophila* viral fraction (1 : 1 dilution from stock as indicated to 2^−1^) pretreated with 5 *μ*g/ml of UVADEX plus UV for 10 mins. Infected cells were cultured for 3 days. Cells were collected and stained with PI (1 *μ*g/1 × 10^6^ cells) at 4°C for 10 min to determine the percentage of cell survival cells.

Since the method of glutaraldehyde fixation was unsuitable for maintaining *Drosophila* APC function while inactivating viruses in *Drosophila* APCs, we needed to find an alternative method. Previous studies addressing methods for inactivating RNA and DNA viruses have used psoralen treatment and UV irradiation [10, 16]. Psoralen irreversibly binds to virus RNA or DNA when the complex between psoralen and nucleic acid is exposure to UV irradiation. This crosslinking actually prevents virus DNA or RNA replication [17]; we then presumed that UVADEX plus UV treatment of *Drosophila* cells would both inactivate *Drosophila* viral activity and preserve APC function. First, we demonstrated that UVADEX treatment can inactivate both insect viral gp64 gene expression and *Drosophial* viruses. As shown in Figure 2(a) (left panel), baculoviruses were detected by gp64 antibody staining; however, UVADEX treatment inactivates baculoviruses to express gp64 (Figure 2(a), right panel). To determine if UVADEX treatment can inactivate *Drosophila* viruses, we purified *Drosophila* viruses from intact *Drosophila* cells and infected to viral free cell line clone 5-5. The observation is shown visually via microscopy analysis (Figure 2(b)); on the left panel, infected clone 5-5 cells underwent apoptosis while UVADEX treatment inactivates *Drosophila* viruses. This result was further confirmed by propidium iodide (PI) staining (Figure 2(c)). Since *Drosophila* viruses contain a *Drosophila* X virus which is a lytic virus, we developed a viral infectivity assay to infect clone 5-5 cell line using a serial diluted viral fraction. As shown from one individual experiment in Figure 2(d), infected clone 5-5 cells underwent cell death with the increased dose of the viral fraction. In contrast, UVADEX treatment of viral fractions prevented infection of clone 5-5 indicator cells. The repeated experiment showed the same result (data not shown).

### 2.3. UVADEX Crosslinking of *Drosophila* Cell Genomic DNA Prevents Cell Growth

**Figure 3:**
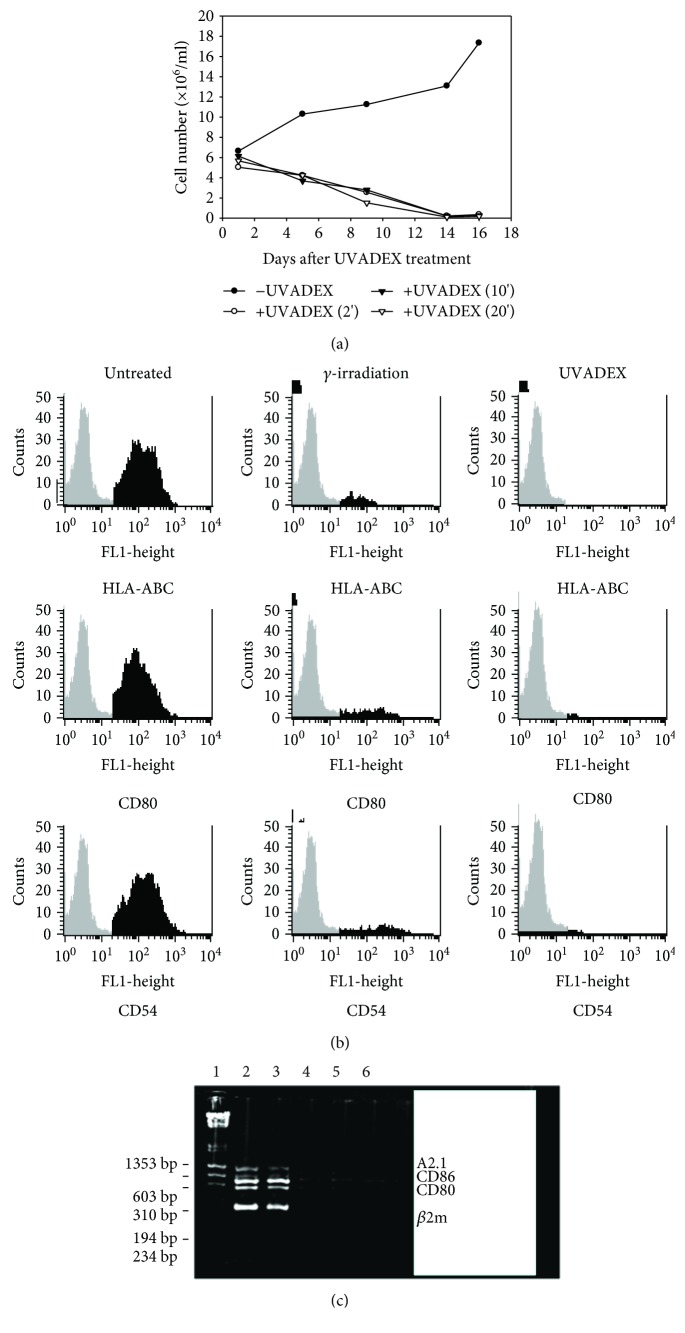
Effect of UVADEX on *Drosophila* cell growth and surface marker expression. (a) *Drosophila* cell line 668 was treated with UVADEX (5 *μ*g/ml) at 4°C for 30 min following UV treatment for 0 min, 2 min, 10 min, and 20 min, respectively. The treated cells were washed completely to remove residual UVADEX and seeded in a 6-well plate at 1 × 10^6^/ml and continually grown for 16 days. Cell survival was monitored by trypan-blue staining. (b) UVADEX treatment completely inactivates *Drosophila* cells. *Drosophila* cell 668 was treated with UVADEX (5 *μ*g/ml) at 4°C for 30 min or *γ*-irradiation for 45 min. The UVDEX-treated cells and *γ*-irradiated cells were continually grown for 16 days, respectively. The cells from two different treatments were collected and stained with FITC-conjugated mAb against HLA-ABC, CD80, and CD54 isotype control antibody and PI at 4°C for 30 min, respectively. Flow cytometry analysis was used to analyze the live cells to determine the expression of HLA-ABC, CD80, and CD54. (c) RNA was isolated from each group of cells above, and RT/PCR was performed using Platinum Taq Polymerase and specific primers for human beta-2-microglobulin, human LFA-3, human CD80, human CD86, and human A2.1. PCR products in each group run on 1% agar. Lane 1, MW marker; lane 2, DNA from *Drosophila* 668 cell; lane 3, DNA from *γ*-irradiated 668 cells; lane 4, DNA from UVADEX-treated 668 cells for 2 min; lane 5, DNA from UVADEX-treated 668 cells for 10 min; and lane 6, DNA from UVADEX-treated 668 cells for 20 min.

Next, we wanted to determine if UVADEX crosslinking affected *Drosophila* viability. The *Drosophila* cell line 668 expressing HLA-A2, B7.1, B7.2, ICAM, and LFA-3 was treated with 5 *μ*g/ml of UVADEX and exposed to UVA long wave light (320–380 nm) for different lengths of time. As shown from one individual experiment in Figure 3(a), UVADEX plus UV treatment inhibited cell growth after as little as 2 minutes of UV exposure (5 J/cm^2^) (open dot line). Two weeks after UVADEX treatment, no live cells remained. In contrast, as an experiment control, *Drosophila* cells not treated with UVADEX remained viable (closed dot line). This result was also repeated from another individual experiment (data not shown). Another widely used method to inactivate cells is *γ*-irradiation treatment. Comparing *γ*-irradiation of *Drosophila* APCs or UVDEX treatment by the FACS analysis of live cell expression of HLA-ABC, CD80, and CD54 from two different treatments demonstrated that UVADEX completely inactivated *Drosophila* cells (Figure 3(b), right channel) while the treatment from *γ*-irradiation did not inactivate *Drosophila* cells completely (Figure 3(b), middle channel). The isotype control in this experiment was not changed (gray area). Also, DNA was no longer detectable from UVADEX-treated *Drosophila* APCs (Figure 3(c), lanes 4–6) indicating that UVDEX treatment of *Drosophila* APCs may crosslink *Drosophila* DNA and affect the DNA implication in PCR. The result indicated that UVDEX treatment of *Drosophila* cells completely abolishes transcription and translation of key molecules such as HLA-A2, CD80, and CD86, which may prevent transmission of xenogenic viruses. Thus, UVADEX treatment ensures the safety of *Drosophila* cells as APCs by inactivating *Drosophila* viruses. In essence, UVADEX treatment changes *Drosophila* cells into cellular vesicles, and thus, the definition of a xenotransplantation product no longer applies.

### 2.4. UVADEX-Treated *Drosophila* Cells Retain APC Function

**Figure 4:**
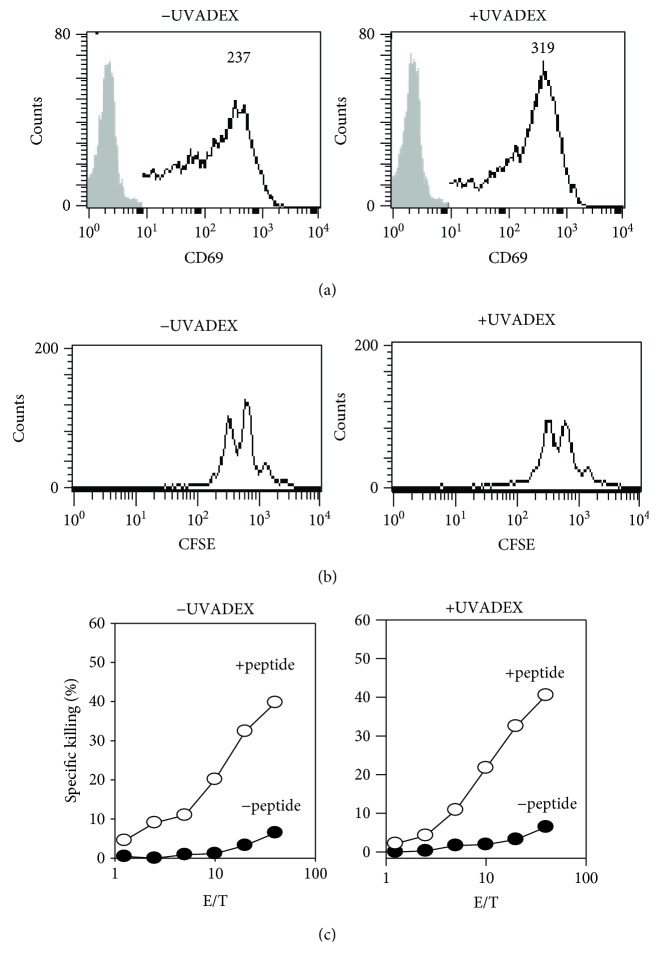
UVADEX-treated *Drosophila* cells retain APC function. (a) CD8 T cells retained CD69 expression when activated by UVADEX-treated *Drosophila* APCs. Purified CD8 T cells from 2C transgenic mice were cultured with APCs (Fly/L^d^/B7.1/ICAM) at 37°C for 4 h. The cultured cells were collected and stained with anti-CD69-FITC mAb at 4°C and isotype control antibody for 30 minutes and analyzed for CD69 expression by FACS analysis. Left panel: CD8 T cells were stimulated by nontreated APCs. Right panel: CD8 T cells were stimulated by UVADEX-treated APCs. The number in the panel indicated the mean fluorescence intensity by FACS. (b) UVADEX-treated APCs stimulate the proliferation of CD8 T cells as efficiently as untreated APCs. CFSE-labeled CD8 T cells purified from 2C transgenic mice were cultured with APCs in the presence of 10 *μ*M of QL9 peptide at 37°C for 2 days. The cultured cells were collected and stained with PE-conjugated anti-mouse CD8 mAb at 4°C for 30 min. CD8-positive cells were gated from FACS and further analyzed for their green fluorescence intensity. (c) CD8 T cells stimulated with UVADEX-treated APCs retain their cytolytic function. CD8 T cells purified from 2C mice were stimulated as described in Figure 1(c). 2C CD8 T cells were used in ^51^Cr release assay with RMAS^Ld^ target cells.

Next, we wanted to address the question whether UVADEX treatment altered APC function. To answer this question, CD8 T cells were purified from 2C TCR transgenic mice and stimulated with UVADEX-treated or nontreated *Drosophila* APC Fly/L^d^/B7.1/ICAM. We analyzed T cell activation, proliferation, and CTL activity to access APC function. A known marker of CD8 T cell activation is the up regulation of cell surface CD69 expression. Thus, we monitored CD69 expression over time of CD8 cells activated with either UVADEX-treated or nontreated APCs. As shown in Figure 4, similar CD69 expression was observed. To access proliferative capacity, CFSE-labeled CD8 T cells were stimulated with treated or nontreated APCs for two days. The results showed that UVADEX treatment does not alter T cell proliferation (Figure 4(b)), as demonstrated by equal CFSE dilution between treated or nontreated groups. To confirm if the proliferation of T cells is an antigen-specific response, CFSE-labeled CD8 T cells were stimulated with treated or nontreated another *Drosophila* APC Fly/D^b^/B7.1/ICAM for two days. The results showed that the stimulated T cells were not proliferated at all (data not shown). Finally, we tested the ability of treated and nontreated *Drosophila* cells to generate CTLs. The results shown in Figure 4(c) demonstrated that T cells stimulated by treated or nontreated APC have similar cytotoxicity. All these repeatable data (data not shown) indicate that UVADEX treatment does not affect *Drosophila* APC function in our experimental condition since we have shown UVDEX-treated *Drosophila* APCs still partially survive and will not completely die until two weeks after treatment (Figure 3(c)).

### 2.5. Expansion of Antigen-Specific CD8 T Cells by UVADEX-Treated APCs

**Figure 5:**
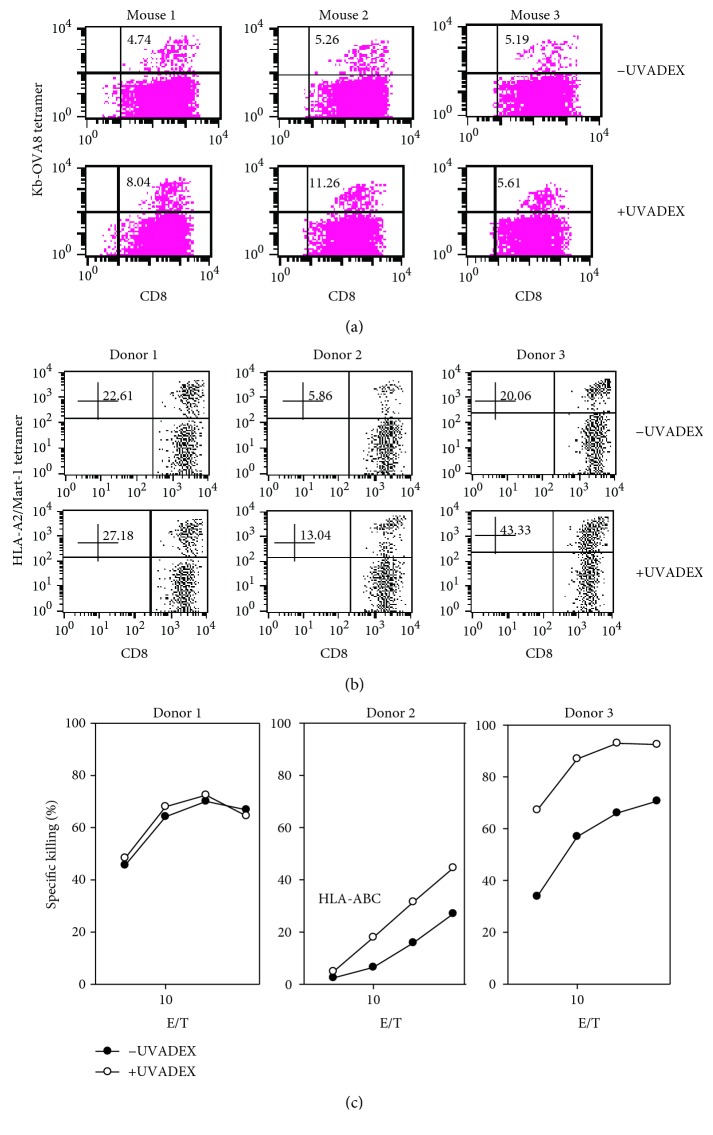
The effect of UVADEX-treated APCs on generation of antigen-specific CTLs from both mice and human. (a) CD8 T cells purified from pooled B6 mice spleen and lymph node cells were stimulated in 24-well plates with OVA8-peptide-loaded and UVADEX-treated or nontreated APCs (Fly/K^b^/B7.1/ICAM). Cells were prepared as described in Figure 1(c). At day 9, cells were collected and stained with OVA/K^b^-specific tetramer-PE or D^b^-GagL-specific tetramer and FITC-conjugated anti-mouse CD8 mAb at room temperature for 30 min. FACS analysis was used to determine the number of OVA-specific CTLs. (b) Purified human CD8 T cells from HLA-A2-positive donors were cultured with Mart-1-peptide-loaded APCs or UVADEX-treated Mart-1-peptide-loaded APCs at 37°C, 5% CO_2_ for 5 days. Human IL-2 and IL-7 were added for further culture. The activated CD8 T cells were restimulated twice at day 7 and day 15 with non-CD8 adherent cells in PBMC from the same donor in the presence of antigen. The antigen-specific CD8 T cells were identified by tetramer staining to determine the number of antigen-specific CTLs. (c) CTLs generated from human donors described in 5b were assayed for cytolytic activities. ^51^Cr-labeled T2 cells were loaded with Mart-1 peptide or without Mart-1 loading and incubated with CTLs generated by UVADEX-treated or nontreated APC stimulation at 37°C, 5% CO_2_ for 4 h. The culture supernatant was collected for the determination of ^51^Cr release.

Tetramer staining of the TCR on the surface of CTLs reveals the antigen-specific nature of the T cell. The percentage of peptide-specific CTLs can be enumerated by this method and allows the determination of the specificity of the ex vivo stimulated T cell preparation. To determine if UVADEX treatment alters the generation of antigen-specific CTLs, the percentage of tetramer-positive CTLs generated in the presence of UVADEX-treated or nontreated APCs was assessed.  Figure 5(a) shows that mouse CTLs generated in the presence of UVADEX-treated *Drosophila* APCs have similar or increased percentage of tetramer-specific CTLs. The control of tetramer staining using D^b^-GagL-tetramer shows total negative (data not shown). To confirm that UVADEX treatment is beneficial for immunotherapy treatment, we tested the ability of *Drosophila* APCs to generate human melanoma-specific CTLs. The naïve human CD8 T cells were stimulated with UVADEX-treated or nontreated APCs loaded with melanoma Mart-1 peptide (Figure 5(b)). The number of Mart-1-specific CD8 T cells was measured by Mart-1/HLA-specific tetramer staining. The control of tetramer staining using HIV-tetramer shows total negative (data not shown). These data showed that the number of antigen-specific T cells stimulated by UVADEX-treated APCs was significantly higher (27.18% versus 22.61%, 13.04% versus 5.86%, and 43.33% versus 20.06%). In addition, CTL cytotoxicity was measured in the end of CTL generation protocol ex vivo by ^51^Cr releasing assay. CTL killing activity is increased significantly in T cells generated by UVADEX-treated APCs from donors 2 and 3 (Figure 5(c), open dot line, subtracted negative control without peptide loading). These data indicate that in addition to retaining APC function, UVADEX treatment can also enhance APC function in some instances (donors 2 and 3).

### 2.6. The Effect of UVADEX-Treated APCs and CpG on Generation of Antigen-Specific CTLs

**Figure 6:**
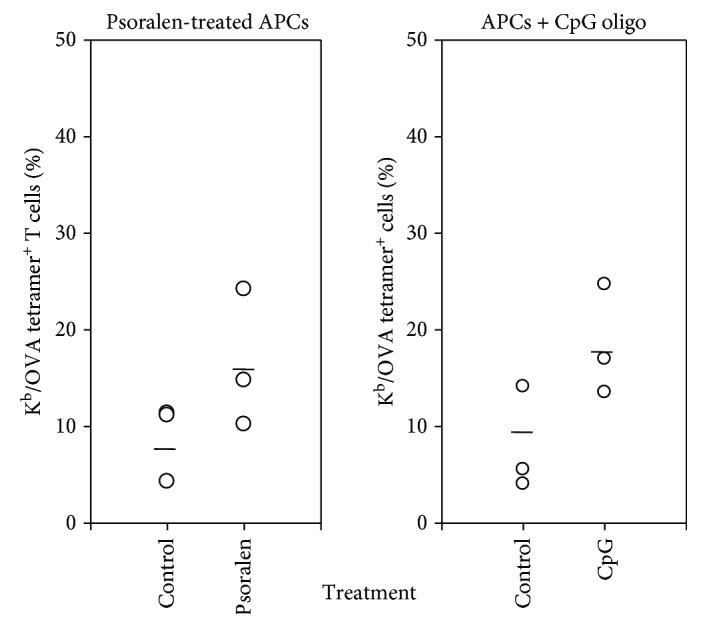
The effect of UVDEX-treated APCs and CpG on the generation of antigen-specific CTLs. CD8 T cells were purified from one pooled B6 spleen and lymph node cells using anti-mouse CD8 beads by Auto*MACS* (Miltenyibiotech), and three mice were used for the experiment. The purified CD8 T cells were activated on 24-well plates with OVA-peptide-loaded and psoralen UV-treated or nontreated APCs (Fly/K^b^/B7.1/ICAM) or nontreated APCs in the presence of 5 *μ*M CpG oligo or 5 *μ*M control oligo (GpC oligo). IL-2 was added at day 3 and day 5, and cells were split at day 7. At day 9, cells were collected and stained with OVA-specific tetramer-PE or D^b^-GagL-specific control tetramer and anti-mouse CD8 mAb-FITC at room temperature for 30 min. FACS analysis of OVA-specific CTLs from CD8-positive cells.

To study the mechanism for enhancing the generation of antigen-specific CTLs by UVADEX treatment, we assume that DNA or RNA/psoralen complexes by UVADEX treatment may mimic CpG DNA structure to activate Toll-like receptors on CD8 T cells. To address our assumption, we compared the effect of UVADEX-treated APCs and CpG on the generation of antigen-specific CTLs. CD8 T cells were stimulated by APCs, UVADEX-treated APC, or APC in the presence of CpG or control oligo GpC for 9 days, and the number of antigen-specific T cells was determined with tetramer staining (Figure 6). The means (horizontal bars) showed the significant statistics, and control tetramer staining showed total negative (data not shown). The data indicated that both CpG treatment and UVDEX treatment result in the increased percent of OVA tetramer-positive cells indicating an enhancement in generating more antigen-specific T cells.

### 2.7. MyD88-Dependent Enhancement of Activation of Antigen-Specific CTLs by UVADEX-Treated APC

**Figure 7:**
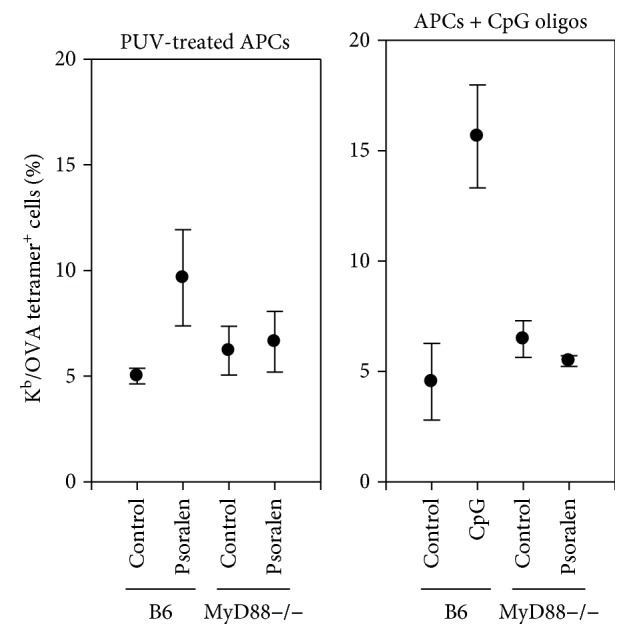
MyD88-dependent enhancement of the activation of antigen-specific CTLs by UVADEX-treated APC. CD8 T cells were purified from one pooled B6 spleen and lymph node cells or one MyD88−/− mouse spleen and lymph node cells by Auto*MACS* (Miltenyibiotech), respectively. Three B6 mice and three MyD88−/− mice were used for the experiment. The purified CD8 T cells were activated on 24-well plates with OVA-peptide loaded and UVADEX-treated APCs (Fly/K^b^/B7.1/ICAM) or UVDEX nontreated APCs (control for psoralen treatment) or nontreated APCs in the presence of 5 *μ*M CpG oligo or 5 *μ*M control oligo (GpC oligo). IL-2 was added at day 3 and day 5, and cells were split at day 7. At day 9, cells were collected and stained with OVA-specific tetramer-PE and or D^b^-GagL-specific control tetramer and anti-mouse CD8 mAb-FITC at room temperature for 30 min. FACS analysis of OVA-specific CTLs from CD8-positive cells.

Since MyD88 is an adapter molecule that is involved in the TLR signaling pathway and TLR-9 binding to CpG is MyD88 dependent, we further confirmed that the enhancement of the activation of antigen-specific CTLs by UVADEX-treated APC is also MyD88-dependent (Figure 7). The error bars showed the significant statistics, and control tetramer staining showed total negative (data not shown). This result showed that no enhancement in generating antigen-specific CD8 T cells from MyD88−/− mice both in UVADEX-treated APC and in the presence of CpG oligo comparing to CpG and UVADEX treatment effect on CTL generation from normal mice, indicating the role of CpG and UVADEX-treatment to generate more antigen-specific CTLs, may be through TLR-9.

## 3. Discussion

*Drosophila* APCs have been demonstrated as effective antigen-presenting cells to stimulate naïve CD8 T cells to generate CTLs in a clinical trial. To reduce the risk of *Drosophila* viral transmission during CTL generation and transfusion, inactivation of *Drosophila* viruses to get a safety CTL product is a primary concern. The most commonly used methods for viral inactivation are solvent detergent and heat inactivation [18]. However, these approaches have been shown to oblate APC function (data were not shown). We have developed an alternative method using psoralen UV derivatives and long-wave ultraviolet light to inactivate viral nucleic acid inside antigen-presenting cells and eliminate their infectivity, while leaving APC function intact. The psoralen photoreaction provides a potential method for inactivating both known and unknown viruses in active products. Brockstedt et al. developed a class of vaccines by psoralen UV technology, based on killed but metabolically active (KBMA) bacteria, that simultaneously takes advantage of the potency of live vaccines and safety of killed vaccines [19].

8-MOP (UVADEX) is one of the psoralen derivatives commonly being used for the inactivation of a variety of viruses in a blood product. Using the 8-MOP (300 *μ*g/ml) and UVA treatment (17 mW/cm^2^) for 60 minutes inactivated cell-associated HIV-1 in platelet concentrates [19]. Treatment conditions of UVDEX have been optimized for *Drosophila* APCs to inactivate *Drosophila* viruses while preserving APC functions. In our experiment, 5 *μ*g/ml of UVDEX and 10 minutes (5 J/cm^2^ UVA) of UVA treatment are sufficient to inactivate *Drosophila* viruses (Figure 1(c)). This condition of photoreaction was also sufficient to inactivate baculovirus in an indirect virus detection assay in which Sf9 cells were infected with UVDEX treated or nontreated baculovirus (data not shown). The viral stock obtained from the supernatant of infected cells did not contain infectious virus in UVDEX-treated viral infection where control viral infected Sf9 cells contained a significant amount of PFUs after a single cycle of infection.

For virus inactivation assay, *Drosophila* virus fractions were prepared from a *Drosophila* line containing *Drosophila* viruses and infected to an indicator cell line clone 5-5 that was absent of *Drosophila* lytic virus DXN. 3 ml of viral fractions prepared from 600 × 10^6^*Drosophila* cells was used for infection assay as viral stock solution.

UVDEX treatment of *Drosophila* cells cultured at 27°C did not replicate. Cell counts were negligible after 14 days in culture (Figure 3). The treatment prevented subsequent replication of the *Drosophila* cells, which remain inactive until they are lysed from lack of growth. This treatment makes *Drosophila* cells like empty vesical. The UVDEX-treated *Drosophila* cells were better APCs than untreated *Drosophila* cells. The ability to maintain or even enhance the APC function of *Drosophila* cell line with UVDEX treatment ensures that the *Drosophila* cells are inactivated prior to exposure to human CD8 T cells. This adds a significant safety feature without diminishing the unique stimulation capacity of the novel APC cells.

The CD8 cells which are specifically stimulated by the UVDEX-treated APC grow as efficiently as those stimulated with nontreated APC. Also, the antigen-specific CTLs generated at the end of the ex vivo culturing cycle are greater than those detected with the untreated APCs. What is the possible mechanism for enhancing antigen-specific CTLs by UVDEX treatment? Recently, the roles of Toll-like receptors in immune recognition and regulation have been broadly studied. Thirteen different TLRs have been identified so far, and some of them recognize molecular patterns such as RNA (TLR3, TLR7, and TLR8), DNA (TLR9), or lipopolysaccharide (TLR4) [20–22]. The interaction between TLRs and their ligands mediates the activation of CD4 and CD8 T cell response. Gelman et al. presented evidence that TLR ligands directly enhance the survival of activated CD4 T cells [23]. Peng et al. reported TLR-8 mediated reversal of CD4+ regulatory T cell function [24]. Liu et al. also reported that TLR signals can overcome T cell tolerance and trigger CD8+ T cell immunity in vitro [25]. In addition, TLR 9, a Toll-like receptor was found in a subset of dendritic cells, B cells, and activated CD4 T cells [26]. TLR9 recognizes a specific pattern of nucleotides in the DNA, known as CpG DNA, which is common in bacteria and viruses, but uncommon in human DNA [27]. Using synthetic CpG sequences capable of binding to and activating TLR9, to find an explanation for our observation, we presumed that DNA or RNA/psoralen complexes released from UVDEX-treated *Drosophila* cells may mimic the CpG structure to activate Toll-like receptors on CD8 T cells or some residual non-CD8 cells during CD8 purification to stimulate naïve CD8 T cells. To confirm that, a parallel *in vitro* experiment to compare the effect of UVDEX-treated APCs and CpG on the generation of antigen-specific CTLs was performed. The results showed that both UVDEX-treated APCs and the presence of CpG oligos have an enhancement in generating more antigen-specific T cells (Figure 6). We further demonstrated that this enhancement in generating antigen-specific CTLs by UVDEX-treated APCs is through the MyD88-dependent pathway (Figure 7).

Elevated programmed death-1 (PD-1) expression can inhibit T cell activity and is a potential barrier to achieving persisting and optimal immunity via vaccination. A study showed that peptide vaccination with escalating doses of CpG ODN adjuvant yielded higher magnitudes of CD8(+) T cells with progressively lower PD-1 expression and greater ex vivo function [28]. Another possible mechanism we will include is that DNA or RNA/psoralen complexes released from UVDEX treated *Drosophila* cells may mimic the CpG structure to facilitate the priming of higher magnitudes of CD8(+) T cells associated with lower expression of PD-1. Future studies will focus on defining the mechanism of psoralen in T cell activation.

In conclusion, our results describe a new method for CTL generation using an artificial APC system. *Drosophila* cells are treated with UVDEX plus UVA to prevent cell growth and viral replication. These APCs maintain their ability to generate CTLs, while preventing xenogenic viral transmission. Thus, inactivated UVDEX-treated *Drosophila* cells provide a clinically feasible system for generating antigen-specific CTLs for immunotherapy.

## 4. Materials and Methods

### 4.1. Cell Lines

The *Drosophila* SC2 and insect Sf9 cell lines were purchased from ATCC. The *Drosophila* APC line 668 expresses HLA-A2, B7.1, B7.2, LFA-3, and ICAM molecules made by scientists in Johnson & Johnson Pharmaceutical Research and Development, LLC. *Drosophila* cell line clone 5-5 is a subclone of *Drosophila* APC line 668 which is *Drosophila* viral X-free. *Drosophila* APC lines SC2/L^d^/B7.1/ICAM and SC2/K^b^/B7.1/ICAM were prepared and used as described previously [29]. Both 668 and *Drosophila* APC lines SC2/L^d^/B7.1/ICAM and SC2/K^b^/B7.1/ICAM were cultured in Schneider's *Drosophila* medium (Invitrogen, San Diego) supplemented with heat-inactivated 10% fetal bovine serum (Irvine Scientific) and additives, including 50 U/ml penicillin, 50 *μ*g/ml streptomycin, 2 mM L-glutamine, and 500 *μ*g/ml G418 (Invitrogen, San Diego). Expression of transfected molecules was induced by culture for 48 hours in the presence of 1 mM CuSO_4_. RMAS-L^d^ cells engineered to express H-2^d^ by transfecting RMAS cells with the pcDNA_3_ vector encoding H-2^d^ gene were provided by H. Eisen (Massachusetts Institute of Technology, Boston, MA). Mammalian cells were grown in RPMI-1640 culture medium (Invitrogen, San Diego) supplemented with heat-inactivated 10% fetal bovine serum and additives, including 50 U/ml penicillin, 50 *μ*g/ml streptomycin, and 2 mM L-glutamine. T2 cells profoundly defective in the presentation of endogenously synthesized antigens to CTL due to a deletion of MHC class II-encoded genes for transporters associated with antigen presentation (TAP1/TAP2) were cultured in RPMI-1640 medium supplemental with heat-inactivated 10% fetal bovine serum.

### 4.2. Reagents

Recombinant human interleukins IL-2 and IL-7 were purchased from R&D Systems. FITC-conjugated anti CD69 and phycoerythrin-conjugated anti-CD8 were purchased from BD PharMingen (San Diego, CA). iTAG™ HLA-A2/Mart-1 Tetramer, iTAG HLA-A2/HIV Tetramer HIV, iTAG K^b^/OV, and iTAG D^b^/GagL Tetramer were purchased from Beckman Coulter. UVADEX was generously provided by Dr. David Peritt (J&J, Therakos). Carboxyfluorescein diacetate succinimidyl ester (CFSE) was purchased from Molecular Probes (Eugene, OR). Glutaraldehyde was purchased from Fisher Scientific Co. (50% biological grade). BacPAK™ Baculovirus Rapid Titer Kit was purchased from BD Bioscience.

### 4.3. Peptides and Oligonucleotides

Peptides used in this study were synthesized on Applied Biosystem model 431 A (Foster City, CA) by standard solid phase peptide synthesis. All peptides were purified with C18 reverse phase HPLC. The concentrations of peptides were determined by quantitative amino acid analysis of HPLC. The sequences of peptides used in this study were as follows: QL9 and QLSPFPFDL [30] and Mart-1, AAGIGILTV, and OVA-8 (SIINFEKL).

### 4.4. Mice

Male wild-type C57BL/6J mice and MyD88 knock-out (MyD−/−) mice were obtained from The Jackson Laboratory (Bar Harbor, ME). 2C TCR transgenic mice were maintained on a C57BL/6 background and kept under pathogen-free conditions at the rodent breeding colony at the Johnson & Johnson Pharmaceutical Research and Development (San Diego, CA).

### 4.5. Preparation of Viral Stock

*Drosophila* viral fractions were obtained from *Drosophila* SC cells by sonication of cell pellet and cesium chloride density ultracentrifugation. In detail, 600 × 10^6^ cells were suspended in 0.6 ml insect cell culture media on ice and sonicated for 30 seconds. Cell debris were removed by ultracentrifugation. The collected supernatant was layered on a cushion of cesium chloride density 1.2 (20% *w*/*w* CsCl) at a ratio of 3 volumes of supernatant for one volume of CsCl and ultracentrifuged at 100000 ×g for 4 h. The viral fractions were collected from the bottom of the tube, and the density of fractions was measured. Fraction densities between 1.1 to and were pooled in 3 ml volumes and dialyzed against PBS completely.

### 4.6. UVADEX Treatment of *Drosophila* APCs

1 mM CuSO_4_-induced insect APC cells were harvested and washed with RPMI-1640/5% FCS media three times. Cells were resuspended in RPMI-1640/5% FCS (4 × 10^6^/ml) including UVADEX (5 *μ*g/ml). 3 ml of cells were added to each well in a 6-well plate. The plate was incubated at 4°C for 30 mins followed by 10 mins of UV using a UV exposure device (Therakos, J&J). Following irradiation, cells were collected and washed with MLR (RPMI-1640/10% FCS) medium three times, resuspended in MLR media (1 × 10^6^/ml) and then loaded with 10 *μ*M peptide at RT for 60 mins.

### 4.7. *Drosophila* X Virus Infection Assay

*Drosophila* viral X-free cell line clone 5-5 was infected with *Drosophila* viral fraction. 1 : 1000 diluted *Drosophila* viral stock was pretreated or nontreated with 5 *μ*g/ml of UVADEX for 10 mins. 1 : 1 serial dilution of this pretreated or nontreated viral stock starting at 1 : 1000 was used to infect cell line clone 5-5. After completely washing with culture media, infected clone 5-5 cells were cultured for 3 days. 1 × 10^6^ infected cells were collected and stained with PI (Sigma, 1 *μ*g/1 × 10^6^ cells) at 4°C for 10 min. Cell survival was monitored via flow cytometry.

### 4.8. UVADEX Treatment of *Drosophila* APCs

*Drosophila* cells were treated with UVADEX (5 *μ*g/ml) at 4°C for 30 min followed by UV treatment for 0 min, 2 min, 10 min, and 20 min, respectively. The treated cells were washed completely to remove residual UVADEX and seeded in a 6-well plate at 1 × 10^6^/ml and cultured for 16 days. The number of live cells was counted at days 1, 5, 9, 14, and 16 by trypan-blue staining. The UVADEX-treated cells were collected at day 16 and used for DNA extraction, and one aliquot of cells was induced at day 15 and stained with FITC-conjugated mAbs against HLA-ABC, CD80, and CD54 by FACS analysis at day 16.

### 4.9. Microtiter Assay for Baculovirus Detection

Sf9 cells were infected with stock virus dilution (BD BacPAK Baculovirus Rapid Titer Kit) and were then treated with UV irradiation following psoralen treatment. Cells were washed completely and grown at 28°C for 4 days. The culture supernatant was collected from growing cells (Passage One virus stock) at day 5 and reinfected to Sf9 cells seeded in a 96-well plate. The plate was fixed by adding methyl cellulose overlay for 48 h. Immunoassay was used to detect the virus by gp64 antibody staining at day 7. Stained foci of infection in the highest dilution wells using light microscopy were counted.

### 4.10. CD8 T Cell Activation and Proliferation Measurement

Purified CD8 T cells (1 × 10^6^/well) from 2C transgenic mice were cultured with APCs (1 × 10^6^/well) expressing L^d^, B7.1, and ICAM-1 at 37°C. Cells were collected at the indicated time points. For the analysis of surface CD69 expression, the cultured cells were collected and stained with FITC-conjugated anti-CD69 mAb at 4°C for 30 minutes and analyzed on a FACScan (BD, California). Cell proliferation was assayed by CFSE labeling. Briefly, 1 ml of 1 × 10^6^ CD8 T cells was labeled in 5 *μ*M CFSE at 37°C for 8 mins and blocked immediately with FCS (2% final). Labeled cells were spun down and washed twice with PBS/2% FCS. CFSE-labeled 2C CD8 T cells (1.0 ml at 1 × 10^6^/ml) were cultured with 1 ml of 10 *μ*M of QL9 peptide preloaded APC (1 × 10^6^/ml) expressing L^d^, B7.1, and ICAM-1 at 37°C for 2 days. The cultured cells were collected and analyzed by flow cytometry.

### 4.11. Mouse CTL Preparation and Cytotoxic Assay

CD8 T cells were purified from lymph nodes of 2C transgenic mice using a positive selection MACS system (Miltenyi Biotec). Purified CD8 T cells from 2C transgenic mice were cultured with APCs (SC2/L^d^/B7.1/ICAM) preloaded with 10 *μ*M QL9 peptide in a 12-well plate at 37°C, 5% CO_2_. 20 U/ml of IL-2 was added at day 3. CD8 T cells were expanded at day 5 and day 7, and CTL activity was measured by ^51^Cr release assay using RMAS-L^d^ as target cells at day 9. To prepare targets, RMAS-L^d^ cells were labeled with ^51^Cr (100 *μ*Ci/1-2 × 10^6^ cells) at 37°C for 60 min. After labeling, the cells were thoroughly washed and resuspended in RPMI medium with 10 *μ*M QL9. The CTL and labeled targets were coincubated at 37°C, 5% CO_2_ for 4 h, and the specific lysis was calculated as previously described [31].

### 4.12. Effects of UVADEX-Treated APCs on the Generation of Antigen-Specific Mouse CTLs

Purified mouse CD8 T cells were incubated in 24-well plates with OVA-peptide-loaded and UVADEX-treated APCs (SC2/K^b^/B7.1/ICAM). 20 U/ml of IL-2 was added at day 3 and day 5. Cells were split at day 7. The expanded cells were collected and stained with OVA-specific K^b^-Tetramer-PE at room temperature for 30 min on day 9. FACS analysis of OVA-specific CTLs was used to determine the percentage of antigen-specific CTLs in culture.

### 4.13. Generation of MART-1-Specific Human CTLs with Untreated or UVADEX-Treated APCs

*Drosophila* APC line 668 was treated with UVADEX as described above. The APCs were then loaded with 10 *μ*M of Mart-1 peptide at room temperature for 4 h. Human CD8 T cells were purified from HLA-A2 positive PBMC and cultured with Mart-1-peptide-loaded APCs or UVADEX-treated Mart-1-peptide-loaded APCs at 37°C, 5% CO_2_ for 5 days. Human IL-2 (20 U/ml, R&D) and IL-7 (30 U/ml, R&D) were added at day 5 for further culture. The activated CD8 T cells were restimulated twice at day 7 and day 15 with non-CD8 adherent PBMCs from the same donor in the presence of Mart-1 peptide. The number of antigen-specific CD8 T cells was identified by Mart-1/HLA-A2 tetramer (Beckman Coulter, CA) staining at day 19.

### 4.14. Determination of Antigen Specificity by Tetramer Staining

MHC tetramer (10 *μ*l) was mixed with the preparation containing the CD8 T cells (10^6^ cells in 100 *μ*l of FACS buffer). The reagent and cells were incubated at RT for 30 minutes followed by washing in PBS and a low-speed spin (400 ×g for 5 minutes). The cell pellet was resuspended in FACS buffer (500 *μ*l) and immediately read on a FACScan flow cytometry machine.

### 4.15. Human CTL Cytotoxic Assay

CTL was generated as described above. CTL assay was determined using T2 target cells at day 19 of CTL generation protocol. T2 cells were labeled with ^51^Cr (100 *μ*Ci/1-2 × 10^6^ cells) at 37°C for 60 min. After labeling, the cells were thoroughly washed and resuspended in RPMI medium with or without 10 *μ*M Mart-1 peptide at RT for 1 h. ^51^Cr-labeled target cells were incubated with CTL in different E/T ratios at 37°C, 5% CO_2_ for 4 h; the culture supernatant was collected and counted for the calculation of specific lysis.

### 4.16. *γ*-Irradiation Treatment of *Drosophila* APCs

1 mM CuSO_4_-induced APCs were harvested and washed with *Drosophila* cell culture medium three times. The washed cells were irradiated at 10000 rads using a Gammacell 1000 *γ*-irradiator machine (Nordion International Inc.). Following irradiation, cells were collected and washed with insect medium three times, resuspended in culture medium (1 × 10^6^/ml), and then cultured for 40 days. The *γ*-irradiated cells were collected at day 40 for DNA extraction, and one aliquot of cells was induced one day before and stained with FITC-conjugated mAbs against HLA-ABC, CD80, and CD54 by FACS analysis.

### 4.17. *Drosophila* DNA Determination by RT/PCR

DNA was isolated from nontreated, UVADEX-treated, or *γ*-irradiation-treated *Drosophila* cells using the Qiagen Blood Amp DNA Kit. A total of 400 ng of DNA from each sample was analyzed by PCR using Platinum Taq Polymerase and vector-specific primers including human beta-2-microglobulin, human LFA-3, human B7-1 (CD80), human B7-2 (CD86), and human A2.1. The PCR protocol consisted of a single stage and 30-cycle amplification using the GeneAmp PCR system 9700 Thermal Cycler. Amplified PCR products including human beta-2-microglobulin (479 bp), human LFA-3 (817 bp), human B7-1 (CD80, 965 bp), human B7-2 (CD86, 1098 bp), and human A2.1 (1207 bp) were loaded on 1% agarose gel.

## Data Availability

The data used to support the findings of this study are available from the corresponding author upon request.
